# Towards an integrated approach for understanding glia in Amyotrophic Lateral Sclerosis

**DOI:** 10.1002/glia.24622

**Published:** 2024-09-25

**Authors:** Stanislaw Majewski, Pierre Klein, Séverine Boillée, Benjamin E. Clarke, Rickie Patani

**Affiliations:** ^1^ Department of Neuromuscular Diseases, Queen Square Institute of Neurology University College London London UK; ^2^ The Francis Crick Institute London UK; ^3^ Sorbonne Université, Institut du Cerveau—Paris Brain Institute—ICM, Inserm, CNRS, APHP Hôpital de la Pitié‐Salpêtrière Paris France

**Keywords:** ALS, glia, multiomics, omics, astrocyte, microglia, oligodendrocyte

## Abstract

Substantial advances in technology are permitting a high resolution understanding of the salience of glia, and have helped us to transcend decades of predominantly neuron‐centric research. In particular, recent advances in ‘omic’ technologies have enabled unique insights into glial biology, shedding light on the cellular and molecular aspects of neurodegenerative diseases, including amyotrophic lateral sclerosis (ALS). Here, we review studies using omic techniques to attempt to understand the role of glia in ALS across different model systems and post mortem tissue. We also address caveats that should be considered when interpreting such studies, and how some of these may be mitigated through either using a multi‐omic approach and/or careful low throughput, high fidelity orthogonal validation with particular emphasis on functional validation. Finally, we consider emerging technologies and their potential relevance in deepening our understanding of glia in ALS.

## Main Point

A graphical summary depicting the past and future of ALS glial research, which will require, alongside the application of molecular and cellular techniques, the utilization of more conventional and emerging omics technologies in addition to dataset integration, AI driven analysis techniques and multi‐omics approaches.

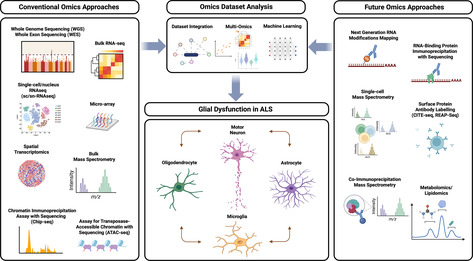

## 1 INTRODUCTION

**FIGURE 1:**
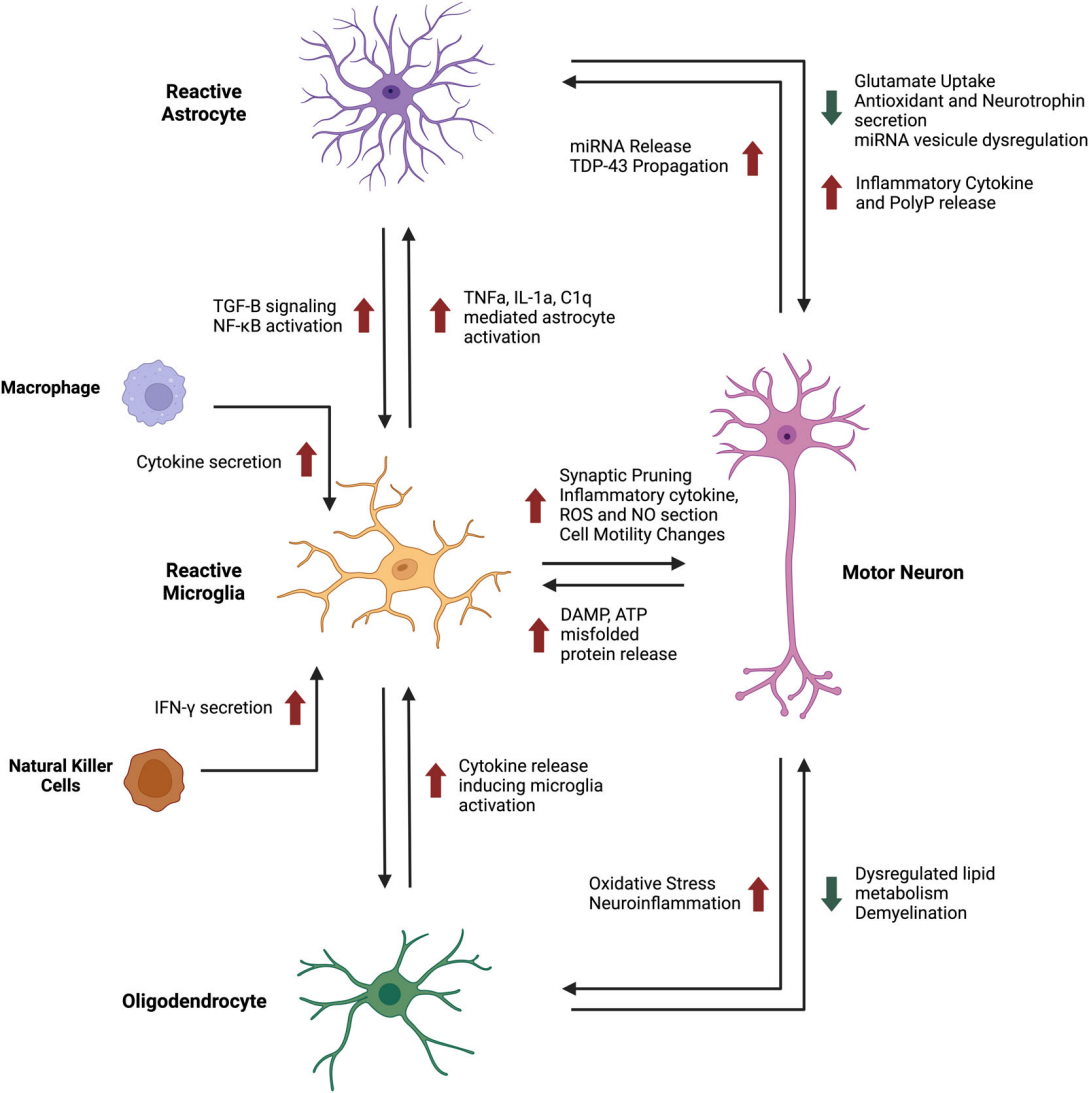
Diagram of the main established roles of glial cells in amyotrophic lateral sclerosis (ALS) pathology. This depicts a reference point for some of the general principles of intercellular relationships that have been studied, mainly using traditional low throughput, candidate based and hypothesis‐driven approaches. ATP: adenosine triphosphate, DAMP: damage‐associated molecular pattern, NO: nitric oxide, ROS: reactive oxygen species.

Glia in the central nervous system (CNS) include astrocytes, oligodendrocytes and microglia, each of which play crucial roles in neuronal homeostasis. Accumulating evidence suggests that dysfunction in each of these cell types, in addition to aberrant interactions between glia and motor neurons, summarised in Figure 1, contribute to amyotrophic lateral sclerosis (ALS) (Patani et al., 2023; Vahsen et al., 2021). ALS is a fatal and currently incurable neurodegenerative disease characterized by upper and lower motor neuron degeneration, resulting in paralysis and death typically 2–5 years after diagnosis (Goutman et al., 2022).

While most cases of ALS have no known genetic cause, more than 30 genes have been linked to ALS with the most common causative mutations identified in *C9orf72* and *SOD1*, with rarer mutations occurring in a number of other genes such as *TARDBP* and *FUS*, most of which are ubiquitously expressed (Akçimen et al., 2023).

Several mouse models of ALS have been established that have deepened our current understanding of glial involvement (De Giorgio et al., 2019; Fisher et al., 2023). The first mouse model of ALS, the *SOD1* mutant transgenic mouse, which recapitulates motor neuron death, muscle atrophy and reduced lifespan, has been instrumental in deciphering a role for glia in cellular pathomechanisms. Early evidence for the contribution of glial cells to ALS pathology came from a study which used mice that contained mixtures of wildtype and *SOD1* mutant expressing cells, referred to as “chimeric” mice (Clement et al., 2003). Chimeras with a higher proportion of non‐neuronal cells expressing mutant *SOD1* had more extensive motor neuron death and a shorter lifespan. Cell type‐specific deletion of mutant *SOD1* from microglia (Boillée et al., 2006), astrocytes (Yamanaka et al., 2008) and oligodendrocytes (Kang et al., 2013) demonstrated their individual roles in contributing to disease in mutant *SOD1* mice. Furthermore, several studies have shown that *in vitro* co‐culture of *SOD1* mutant mouse astrocytes (Di Giorgio et al., 2008; Nagai et al., 2007), microglia (Frakes et al., 2014) and oligodendrocytes (Ferraiuolo et al., 2016) confers toxicity to motor neurons. Several mouse models expressing ALS‐related genes including *FUS*, *TARDBP*, *VABP*, *VCP* and *UBQLN2* at physiological levels have recently been developed, which will provide further understanding for the role of glia in ALS (De Giorgio et al., 2019; Zhu et al., 2023).

The advent of human *in vitro* models of glia have enabled further study of the primacy and cell type‐specific nature of glial pathological events (Franklin et al., 2020). Human induced pluripotent stem cell (hiPSC) derived models from ALS patients have demonstrated cell autonomous changes in astrocytes (Birger et al., 2019; Hall et al., 2017; Madill et al., 2017; Serio et al., 2013; Taha et al., 2022; Ziff, Clarke, et al., 2021), microglia (Banerjee et al., 2023; Kerk et al., 2022; Vahsen et al., 2023) and oligodendrocytes (Ferraiuolo et al., 2016; Livesey et al., 2016). Furthermore, transdifferentiation of ALS patient fibroblasts into astrocytes, which maintains the age‐related phenotypes of these cells, have also displayed dysfunction and the ability to induce non‐cell autonomous motor neuron death (Allen et al., 2019; Meyer et al., 2014; Varcianna et al., 2019).

In this review, we will discuss how advances in omics technology have the potential to transform our current understanding of glial cells in ALS. Widespread access to sequencing technologies is catalyzing our understanding of the complexity of different glial reactive states and populations, first in bulk RNA sequencing and more recently using single‐cell technologies, which have aided the understanding of cell type‐specific responses and cell–cell interactions. Furthermore, spatial transcriptomics permits insight into the principles governing spatial organization within individual glial cells. In addition, we will discuss nascent multi‐omic technologies for probing different layers of gene regulation, including chromatin accessibility, transcription, RNA localization, translation, and metabolomics.

**FIGURE 2:**
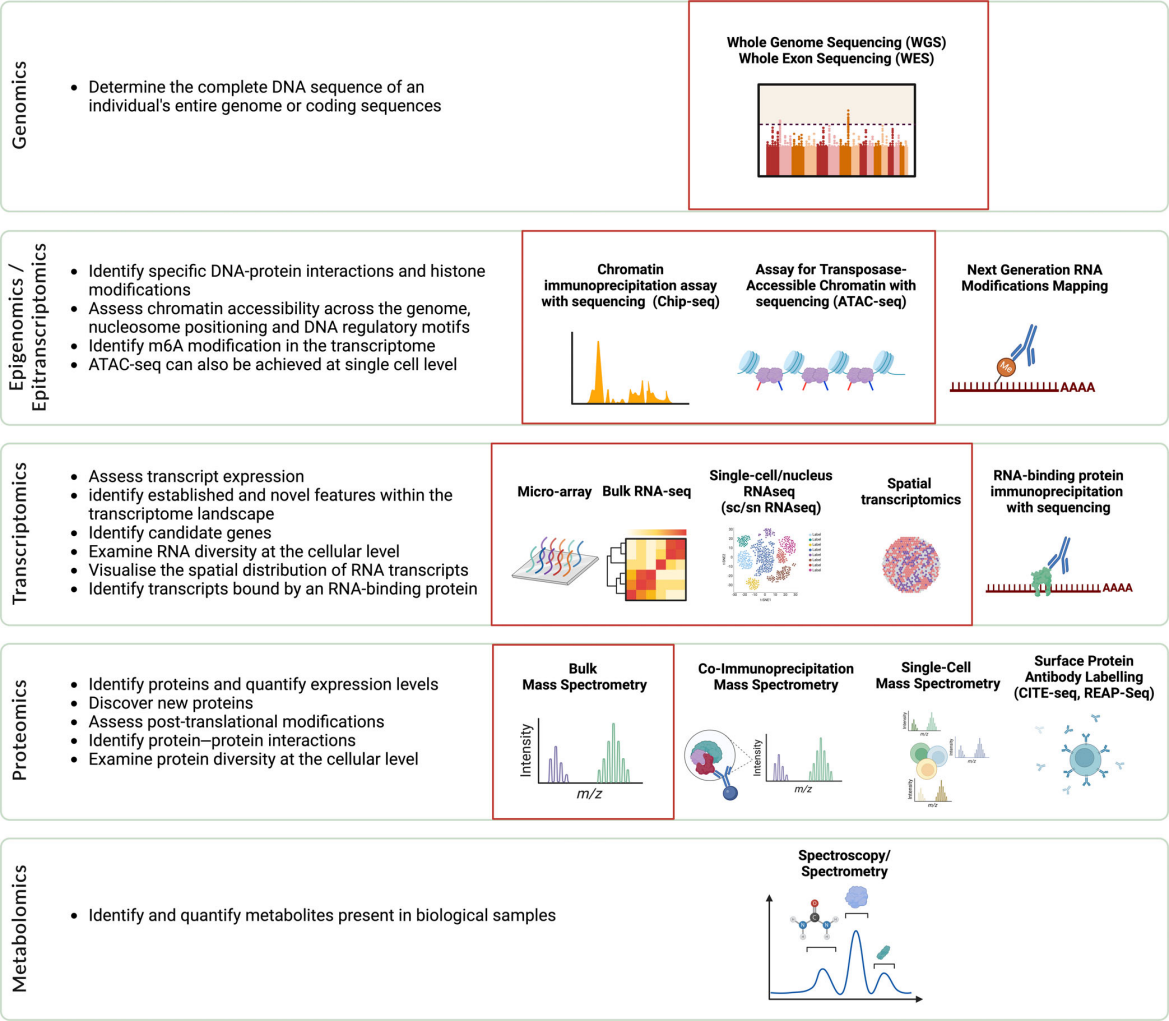
The existing omics fields and various associated technologies. Strategies previously applied in the study of amyotrophic lateral sclerosis (ALS) glia are denoted within red squares. A brief description accompanies each approach and conveys their discovery potential in future studies.

“Omics” is a term which is used to describe various fields of research focused on investigating the entirety of a specific type of biomolecule (see Figure 2). These fields span the examination of the genome (genomics) and its modifications (epigenomics), cellular RNAs (transcriptomics) along with their modifications (epitranscriptomics), proteins (proteomics) and their interactions (interactomics: protein–protein, protein–DNA, protein–RNA), and the study of metabolites (metabolomics). In the biomedical field, these omic methods have instigated a profound transformation in our discovery potential, paving the way for innovative personalized (or ‘precision’) therapeutic approaches. When applied to ALS, these approaches raise the prospect of deciphering the disease's intricate nature, spanning from its inciting pathophysiological mechanisms to those driving progression and advanced stages of disease.

## 2 CONVENTIONAL OMICS APPROACHES TO STUDYING GLIA

**FIGURE 3:**
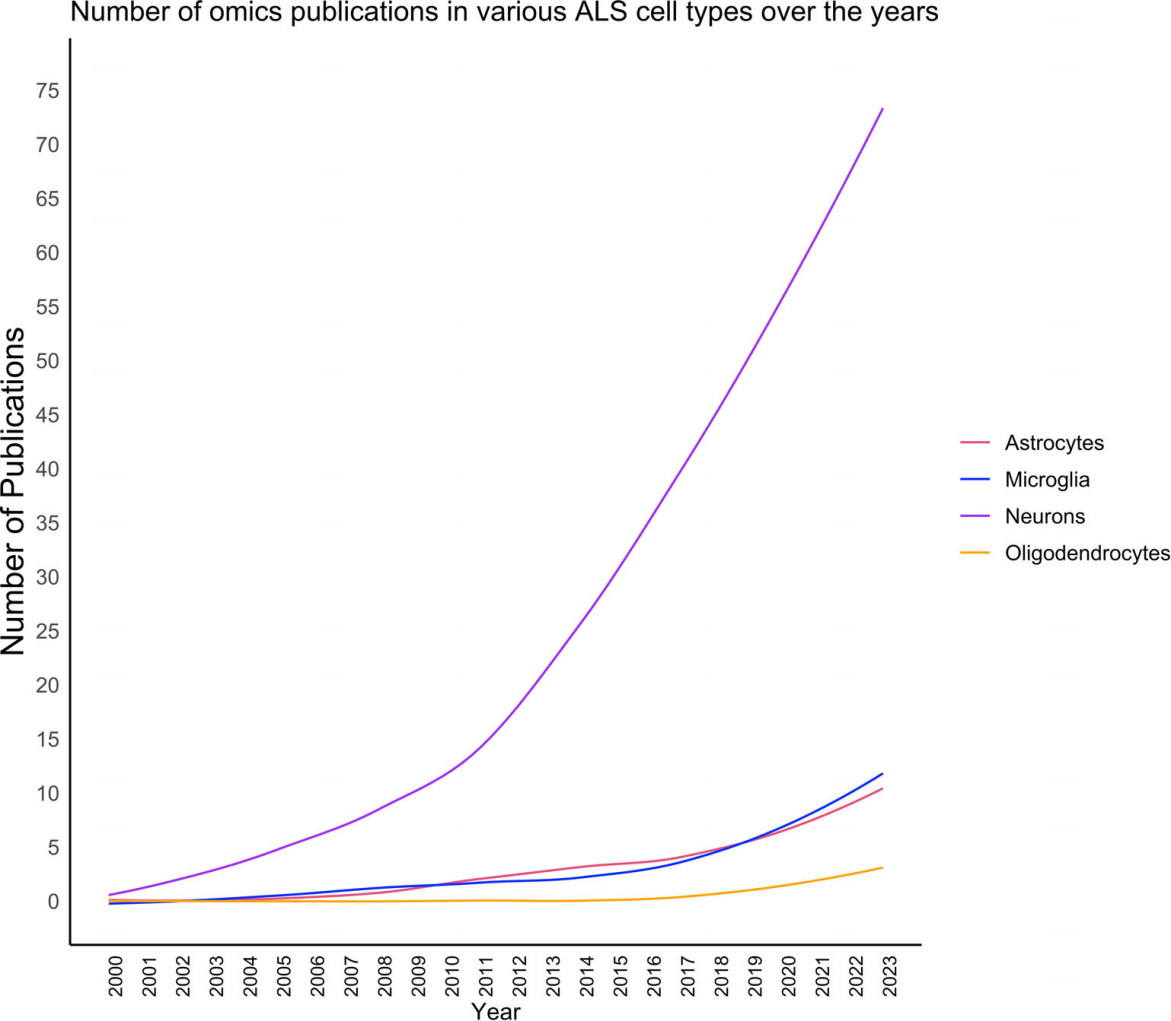
Pubmed search of the number of omics publications by cell type since 2000. Solid lines highlight smoothed trends, providing a clear visualization of the overall research trajectory for each cell type.

The variation across scales, including the clinical presentation of patients, site of onset, disease rate of progression, response to therapy and cell type involvement, among a plethora of other factors, has resulted in lack of a unified understanding of the pathophysiology of ALS. A wide array of glial perturbations have been identified, aided through the increased applications of omics, most notably bulk transcriptomics. Over the last two decades, there has been an increase in the number of publications employing omics methods, but also a diversification in focus on a wider variety of glial cell types and the cellular interactions between glia and neurons. Relative to neurons, the contributions to ALS pathogenesis, and the application of omics techniques to address this, of astrocytes, microglia, oligodendrocytes and even more so other non‐neuronal cell types, remain less well characterized and understood (see Figure 3).

Pubmed query was as followed '"transcriptomics" OR "proteomics" OR "metabolomics" OR "genomics" OR "epigenomics" OR "RNA‐sequencing" OR "Mass Spectrometry" OR "Chromatin Immunoprecipitation Sequencing" OR "ChIP‐Seq" OR "ATAC‐Seq" OR "Assay for Transposase‐Accessible Chromatin Using Sequencing" OR "microarray" OR "single‐cell RNA‐seq" OR "single‐nuclei RNA‐seq" OR "spatial transcriptomics" OR "single‐cell Mass spectrometry" OR "Whole Genome Sequencing" OR "Whole Exome Sequencing" OR "lipidomics" OR "exome sequencing") AND “CELL TYPE” AND "Amyotrophic Lateral Sclerosis”’, with “CELL TYPE” representing either neurons, microglia, oligodendrocytes or astrocytes. Reviews were excluded from this search and the list of publications was manually curated.

### 2.1 Astrocytes

Astrocytes perform numerous homeostatic roles supporting neurons, including the production and uptake of neurotransmitters, blood flow regulation, debris clearance, synaptogenesis among others (Mahmoud et al., 2019; Marina et al., 2020; Peteri et al., 2019; Tasdemir‐Yilmaz & Freeman, 2014). In neurodegenerative diseases such as ALS, astrocytes become reactive, whereby they undergo remodeling in response to injury, disease or infection (Escartin et al., 2021), ultimately resulting in changes to their function. Although the causal timeline of astrocyte reactivity in ALS is unknown, numerous lines of evidence suggest astrocyte reactivity changes can occur both cell‐autonomously but also as a result of changes to the surrounding environment. These include astrocyte‐neuron signaling perturbations (Licht‐Murava et al., 2023; Phatnani et al., 2013; Tripathi et al., 2017; Tyzack et al., 2017), microglia mediated activation (Guttenplan et al., 2020; Liddelow et al., 2017) and astrocyte cell‐autonomous changes during disease progression (Taha et al., 2022). In addition to increased inflammatory processes associated with neurotoxicity, loss of homeostatic function in astrocytes, such as glutamate uptake, has been suggested in ALS models and post mortem ALS tissue (Guo et al., 2003; Lin et al., 1998; Rothstein et al., 1992; Sasaki et al., 2000; Ziff, Clarke, et al., 2021).

#### 2.1.1 Studies predominantly using rodent transgenic models of ALS

Omics techniques have aided our understanding of perturbations potentially involved in astrocyte reactivity changes in ALS using rodent transgenic models, which capture the complex mosaic of the variety of cell types affected in ALS. For example, RNA‐seq analysis following *SOD1* ^*G93A*^ neuron and astrocyte cocultures revealed TGF‐B signaling pathway dysregulation, in agreement with immunohistochemistry of *SOD1* ^*G93A*^ spinal cords where reactive astrocytes were located in close proximity to motor neurons positive for TGF‐BII receptors (Phatnani et al., 2013). Further analysis revealed an increased astrocytic expression of genes involved in secreted glycoproteins, the extracellular matrix, cell growth, migration and cytoskeletal proteins as well as changes in inflammatory responses. Astrocytic TGF‐B secretion has also been found to contribute to motor neuron toxicity by inducing protein aggregation as a result of impaired mTOR mediated autophagy in cocultures of human motor neurons and astrocytes derived from mutant SOD1^*G93A*^ mice (Tripathi et al., 2017).

Aberrant cell signaling changes have also been demonstrated between astrocytes and microglia in ALS. A potential mechanism for harmful astrocyte reactive transformation was demonstrated by crossing *SOD1* ^*G93A*^ mice with a triple knockout mouse line for 3 factors, IL‐1α, TNFα and C1q (TIC) (Guttenplan et al., 2020), known to be secreted by microglia to induce a neurotoxic reactive state in astrocytes (Guttenplan et al., 2020; Liddelow et al., 2017). This led to a reduction in the expression of astrocyte reactivity marker C3, partially rescued motor neuron death and substantially prolonged lifespan of mutant *SOD1* ^*G93A*^ mice (Guttenplan et al., 2020). RNA‐seq of both wildtype and *SOD1* ^*G93A*^ astrocytes isolated by immunopanning and treated with TIC *in vitro*, revealed many genes involved in interferon signaling pathways to be upregulated in mutant conditions. In addition, bulk RNA‐seq of isolated *SOD1* ^*G37R*^ mutant astrocytes at an early disease stage, where muscle denervation had just begun, revealed increase expression of genes involved in inflammation and metabolism, possibly to due to increased activity of nuclear receptors PPAR and LXR (Sun et al., 2015), known to drive transcription of many target genes involved in inflammation (Glass & Rosenfeld, 2000; Hong & Tontonoz, 2008).

The role of gain of toxic astrocyte function in ALS has been further aided though omics techniques. Loss of the endoplasmic reticulum (ER) associated protein membralin has been shown to mediate astrocytic neurotoxic effects through the major glutamate transporter EAAT2 (Jiang et al., 2019). RNA‐seq of motor cortices from astrocyte‐specific membralin knockout mice showed that several dysregulated genes involved in immune responses overlapped with astrocytes isolated from *SOD1* ^*G37R*^ mice. Furthermore, overexpression of membralin in *SOD1* ^*G37R*^ mice prolonged mouse survival.

Numerous genes associated with ALS have been shown to regulate astrocyte reactivity. For example, astrocyte specific *TARDBP* conditional biallelic deletion has been shown to result in increases in *GFAP* immunoreactivity and morphological changes involving increased astrocyte process length (Peng et al., 2020). However, no significant motor neuron death or neuromuscular junction denervation was observed, suggesting that although perhaps contributory, astrocytic TDP‐43 loss of function is not sufficient to cause neurodegeneration. To better understand astrocyte reactivity changes following astrocyte specific TDP‐43 deletion, RNA‐seq of whole spinal cords revealed differentially expressed genes, including previously identified transcriptomic markers of neurotoxic astrocyte reactivity (Liddelow et al., 2017). Gene set enrichment analysis revealed reduced expression of genes involved in homeostatic support functions, including the ensheathment of neurons and axon development. TDP‐43 knockdown in primary rat astrocytes has been further utilized to study the mechanisms implicated in the protein's loss of function (LaRocca et al., 2019). RNA‐seq analyses showed that following TDP‐43 siRNA treatment, genes associated with inflammatory pathways were upregulated, including markers of neurotoxic astrocyte reactivity. Downregulation of genes relating to astrocytic morphology was observed. Further to this, hippocampal astrocytic TDP‐43 cytoplasmic accumulation has been shown to contribute to cognitive decline and memory loss through localized increases in interferon inducible chemokines such as CXCL9 and CXCL11 and their corresponding receptor CXCR3 (Licht‐Murava et al., 2023).

In addition to a vast array of studies addressing ALS astrocyte reactive transformation at the RNA level, proteomics is becoming an increasingly utilized technique within the field. Bulk proteomics of primary cultures has been used to study the effects of angiogenin, a protein associated with neuroprotection (Steidinger et al., 2011; Subramanian et al., 2008), which is released from motor neurons and taken up by astrocytes (Skorupa et al., 2013). Loss of function mutations in the angiogenin gene (*ANG*) have been linked to ALS (Conforti et al., 2008; Paubel et al., 2008). Primary mouse astrocyte cultures treated with angiogenin (Conforti et al., 2008; Paubel et al., 2008; Skorupa et al., 2013) resulted in increased secretion of a number of proteins, including Plau and Tgfbi, which have previously been implicated in neuroprotective functions (Dobolyi et al., 2012; Glas et al., 2007; Tesseur et al., 2017). Analysis of all significantly changed proteins revealed overrepresentation in those primarily related to (i) the extracellular matrix and basement membrane, (ii) binding abilities to chemokine and cytokine receptors and (iii) cellular response to stress and extracellular stimuli. Secretome profiling may be a key resource for not only better understanding and mapping glia proteomic perturbations in ALS, but also for the identification of potential therapeutic targets.

Alongside deciphering disease mechanisms and progression, our understanding of possible astrocytic treatment targets is aided through omics techniques due to their potential to assess a wide range of biomolecules simultaneously. Building upon previous findings implicating increased activation of the Na+/K+ ATPase in astrocyte non‐cell autonomous neurodegeneration (Gallardo et al., 2014), knockdown of *Atp1a2* in primary mutant *SOD1* ^*G93A*^ mouse astrocyte culture, a gene which encodes for the alpha‐2 subunit of the Na+/K+ ATPase protein, resulted in a reduction in SOD1 aggregation, but accelerated disease onset and death (Iyer et al., 2023). RNA‐seq analysis of primary mouse astrocyte cultures and mouse spinal cord samples found common metabolic pathway downregulation, among many other pathway perturbations, possibly responsible for the observed deleterious effects of *Atp1a2* knockdown.

#### 2.1.2 Studies using hiPSC models of ALS or human postmortem tissue

Cell signaling changes have also been demonstrated in human models of ALS. For example, ephrin signaling, a pathway implicated in astrocyte mediated neurogenesis (Ashton et al., 2012) and injury response (Bundesen et al., 2003; Van Hoecke et al., 2012), that has also been suggested to regulate neuroprotective astrocyte reactivity, has been shown to be perturbed in ALS (Tyzack et al., 2017). Bulk RNA‐seq of SOD1^D90A^ mutant iPSC derived astrocytes revealed transcriptional differences in reactivity and proinflammatory genes. Specifically, the EphB1 pathway was identified to be involved in mediating neuroprotective astrocyte reactivity to injured motor neurons through STAT3 signaling. *SOD1* ^*D90A*^ mutant astrocytes were shown to display aberrant cell autonomous EphB1‐mediated responses and impaired STAT3 activation, resulting in a loss of neuroprotective function. Mass spectrometry analysis revealed a number of dysregulated proteins involved in EphB1–ephrin‐B1–STAT3 signaling. However, transcript and protein levels were found to not necessarily always correlate. An example of this was CHCHD2, a mitochondrial protein implicated in stress response (Ruan et al., 2022) and associated with neurodegeneration (Ogaki et al., 2015), increased significantly at the RNA but not protein level in diseased astrocytes.

*C9orf72* mutant human iPSC derived astrocytes have also been shown to undergo transcriptomic perturbations. Changes within cell cycle inhibition and cellular senescence pathways were found, coinciding with neurotoxicity through dysregulation of secreted factors (Birger et al., 2019). Astrocyte secretome changes included lower expression levels of antioxidants *SOD1*, *SOD2* and *GSS*. Human motor neurons treated with astrocyte conditioned medium showed greater reactive oxygen species generation, which may contribute to non‐cell autonomous mechanisms of motor neuron death (Rojas et al., 2015; Cunha‐Oliveira et al., 2020). A variety of other transcriptomic changes have been identified in *C9orf72* mutant hiPSC‐derived astrocytes (Zhao et al., 2020). Analysis indicated upregulation in genes involved in RNA processing in addition to ribosomal large subunit assembly, complement activation, and downregulated genes primarily involved in cell adhesion, chemical synaptic transmission and assembly, sodium and potassium transport and cell‐to‐cell pathways. Possible non‐cell autonomous effects associated with these transcriptomic changes were explored utilizing functional studies involving co‐culturing of healthy motor neurons together with both control astrocytes and mutant *C9orf72* astrocytes. This experiment revealed motor neuron hypoexcitability and significant decreases in resting membrane potentials, peak Na^+^ and K^+^ currents and the proportion of motor neurons able to generate action potentials. Apparent differences in transcriptomic findings between these two studies using *C9orf72* mutant astrocytes may include the differential use of isogenic corrected or healthy control lines and differences in the filtering of differentially expressed genes, among other factors.

*SOD1* ^*D90A*^ and *VCP* mutant ALS iPSC derived astrocytes have also been shown to undergo divergent initial reactive transformation cell‐autonomously (Taha et al., 2022). Differences were found when comparing reactivity markers from transcriptomic data, C3 expression as well as differing basal cytokine secretion levels. While *VCP* mutant astrocytes featured changes in gene ontologies most notably linked to the MHC class II response, *SOD1* ^*D90A*^ mutant astrocytes were characterized by ontologies linked to cell rearrangements and stimulus response. Differences in transcription factor activities inferred from gene expression changes were also found, possibly responsible for the aforementioned gene expression changes. It is possible that the reactivity states converge as the disease progresses, but this initial divergence may ultimately inform genotype‐specific therapeutic approaches.

While different mutant ALS iPSC derived astrocytes have shown relatively little overlap in many specific differentially expressed genes, by analyzing publicly available RNA‐seq data, common dysregulation of processes were found across *C9orf72*, *SOD1*, *FUS* and *VCP* mutants (Ziff, Clarke, et al., 2021). To increase the power and precision in identifying genes that were dysregulated in ALS hiPSC astrocytes, a meta‐analysis of all publicly available ALS iPSC astrocyte datasets was performed. This identified upregulation in genes relating to ER, extracellular matrix, protein metabolism and adhesion and downregulation in genes involved in synaptic and neuronal development. Overlap with these identified genes and pathways was also observed when comparing to control astrocytes treated with inflammatory factors known to be secreted by microglia to induce astrocyte reactivity (Liddelow et al., 2017), suggesting heightened overall basal reactivity levels. Pathway responsive gene analysis (PROGENy) also revealed similarities in increases of TGF‐B signaling, hypoxia, Wnt, and VEGF pathways. Additionally, intron retention (IR), a form of alternative splicing involved in the regulation of gene expression (Jacob & Smith, 2017), has been shown to be reduced in *VCP*, *SOD1* and *C9orf72* mutant hiPSC‐derived astrocytes, correlating with an increased expression of astrocyte reactivity‐related transcripts (Ziff, Taha, et al., 2021). Cumulatively, these studies demonstrate the capabilities of analyzing omics datasets to reveal the complex perturbations responsible for astrocyte reactivity changes observed in ALS.

A variety of omics techniques are additionally being applied to three‐dimensional ALS/frontotemporal dementia (FTD) organoid models. Bulk RNA‐seq analysis of GRN^*−/−*^ organoids containing astrocytes and oligodendrocytes revealed downregulation in a number of astrocytic genes considered to be involved in phagocytosis (de Majo et al., 2023). Patients harboring loss of function granulin (*GRN*) mutations display TDP‐43 proteinopathy (Baker et al., 2006). Functional assays performed on 2D cultures, further revealed perturbed astrocyte synaptosome phagocytosis, mirrored by a higher synaptic density found in GRN^*−/−*^ organoids. *GRN* knockout in both astrocytes and neurons increased pTDP‐43 levels, TDP‐43 extranuclear localization and mis‐spliced STMN2 transcripts containing cryptic exons (CrSTMN2), the latter suggesting loss of TDP‐43 function. Further specific *GRN* knockout in either neurons or astrocytes revealed the ability of *GRN* ^*−/−*^ astrocytes to induce CrSTMN2 increases in *GRN* ^*+/+*^ neurons but not vice versa. When assessed in astrocyte and neuron *GRN* ^*−/−*^ 2D cultures, similarly CrSTMN2 expression was increased. When treated with recombinant PGRN, the protein encoded by *GRN*, CrSTMN2 decreased to similar levels observed in *GRN* ^*+/+*^ cultures, however this was not sufficient to reverse the previously captured phagocytosis deficits. These results suggest cell‐type specific disturbances with relation to TDP‐43 pathology through *GRN* knockout and further emphasize the potential importance of diseased astrocytes in contributing to neuronal death.

Alongside differential gene expression changes, RNA‐seq analysis has determined an enrichment in repetitive element (RE) transcripts following TDP‐43 knockdown, including MER21B, a RE increased in ALS patient post mortem brain samples (Prudencio et al., 2017), potentially due to changes in RNA polymerase II (LaRocca et al., 2019). REs are known to be predisposed to form double stranded RNA (dsRNA) (Cristofari, 2017). Reflecting this, specific antibodies were utilized to identify significant increases in dsRNA, potentially driven by rises in REs. Inhibition of the dsRNA sensor PKR resulted in an amelioration of inflammatory markers in TDP‐43 knockdown astrocytes. This suggests a potential mechanism by which astrocytic TDP‐43 loss of function results in increased activation of inflammatory pathways through dsRNA accumulation activating PKR. Further exploration of RE transcripts, and their relevance to both glial biology, may be of value due to their association with aging (LaRocca et al., 2020) and unclear relationship with *TARDBP* RNA or pTDP‐43 protein levels (Krug et al., 2017; Li et al., 2012).

### 2.2 Microglia

Similarly to astrocytes, a diverse array of reactive changes have been ascribed to ALS microglia. The extent and nature in which dysfunctional microglial reactivity contributes to pathogenesis remains unclear.

#### 2.2.1 Studies predominantly using mouse transgenic models of ALS

Early studies utilizing bulk RNA‐seq of *SOD1* ^*G93A*^ mouse microglia isolated from spinal cord revealed a range of temporal changes in genes associated with both neuroprotection and neurotoxicity (Chiu et al., 2013). ALS mutant microglia displayed transcriptomic signatures featuring lysosomal genes. However, substantial differences in microglial reactivity have been found across ALS models and patient samples (Spiller et al., 2018). The use of a mouse model, rNLS8, to induce formation of TDP‐43 aggregates, resulted in only modest changes in microglial density and no morphological changes following significant motor neuron death, perhaps due to the neuronal specific expression of TDP‐43 with a defective nuclear localization signal.

This differs substantially from *SOD1* mutant mice, where mutant protein is ubiquitously expressed leading to a very different progression of disease, and prominent microglial reactivity changes are present either at or before disease onset (Boillée et al., 2006; Clement et al., 2003). The suppression of *TDP‐43ΔNLS* upon chronic activation prior to or following motor neuron death suggested that microglial reactivity may be independent of motor neuron death (Spiller et al., 2018). RNA‐seq revealed several differentially expressed genes in *SOD1* mutant mice (Chiu et al., 2013) were upregulated across disease and early recovery stages in rNLS8 mice. However, numerous previously identified homeostatic genes found to be downregulated during neurodegeneration remained unchanged (Krasemann et al., 2017). Results broadly suggest, however, that microglia mediated neuroinflammation during ALS disease progression may be potentially neuroprotective through supporting axonal regeneration.

The contribution of peripheral immune cells to aberrant microglial reactivity and neuroinflammation in ALS is complex, involving multiple cell types including monocytes/macrophages and lymphocytes (Berriat et al., 2023). Microglial responses in *SOD1* mutant mice were shown to be suppressed by transplantation of wildtype hSOD1 overexpressing peripheral macrophages at disease onset (Chiot et al., 2020) by acting directly from the periphery. Time‐resolved bulk RNA‐seq analysis revealed that microglial inflammatory genes associated with neuroinflammation that were increased throughout disease course were suppressed by macrophage transplantation while genes involved in pathways including oxidative phosphorylation and synaptogenesis downregulated during the disease were reversed by wildtype peripheral macrophage transplantation.

C9orf72 has been identified as central to regulating the function of CNS immune cells through a series of landmark studies utilizing mouse lines with loss of C9orf72 (Atanasio et al., 2016; Burberry et al., 2016; McCauley et al., 2020; O'Rourke et al., 2016). Studies utilizing C9orf72^−/−^ have found broad phenotypic overlap, characteristic of an autoimmune disease but not neurodegeneration (Burberry et al., 2016; O'Rourke et al., 2016). It was subsequently shown that the gut microflora modified inflammatory phenotypes and long term survival of mice (Burberry et al., 2020). C9orf72^−/−^ mouse transcriptomes featured several upregulated inflammatory genes, interestingly overlapping with those previously found in C9orf72 expansion carriers, but not sporadic, patient spinal cord samples (Prudencio et al., 2015).

Building upon these findings, *C9orf72* loss in dendritic cells corresponded with an upregulation of type I interferon responsive genes and a variety of cytokines due to diminished STING lysosomal degradation (McCauley et al., 2020). This was reflected in patient sequencing datasets, from both blood and brain tissue (Prudencio et al., 2015), where GSEA found upregulation of interferon signaling in C9‐ALS but not sporadic samples, potentially contributing to the association of ALS patients, including those harboring *C9orf72* mutations, with autoimmune disease diagnoses (Miller et al., 2016; Ralli et al., 2019). The currently underexplored intersection between genes associated with ALS, inflammation and environmental factors, aided by the application of omics technologies and large datasets, holds promise in developing our understanding of the heterogeneity across scales in ALS.

The selective targeting of neuroinflammation and microglial activation has been aided through the application of omics methods to identify potential microRNA targets. For example, ALS microglial microRNA changes have also been found throughout disease progression. MicroRNA miR‐155 was identified as increased in *SOD1* ^*G93A*^ mice from a Nanostring panel of microglial homeostatic and inflammatory genes (Butovsky et al., 2015). miR‐155 has various roles, including within Th17 cell generation (O'Connell et al., 2010) and cytokine secretion (Lu et al., 2014), alongside being implicated in multiple sclerosis (Murugaiyan et al., 2011). Deletion of miR‐155 prolonged survival of *SOD1* mutant mice and resulted in a reduction of proinflammatory genes and significant restoration of microglial genes associated with maturation of phagocytes and chemotaxis. Importantly, these transcriptomic findings were validated functionally as miR‐155 ablation restored their ability to phagocytose dead neurons.

Similarly, RNA‐seq analysis following microglial specific deletion of *Ager*, the gene which encodes for RAGE, which has been shown to be upregulated from spatial transcriptomics of mutant *SOD1* mice and ALS patient spinal cords (Maniatis et al., 2019), led to broadly restorative homeostatic transcriptome changes (MacLean et al., 2021). *Ager* deletion within microglia also affected astrocytes, where reductions in reactivity were inferred through a reduction in GFAP area among other parameters and extended lifespan in male but not female SOD1^*G93A*^ mice. Together, this study indicates the potential of the application of omics to better discern treatment effects but also important cross talk between different glial cell types.

#### 2.2.2 Studies predominantly using hiPSC models of ALS or human postmortem tissue

Human iPSC models of microglia have also been used to examine ALS phenotypes. One of the first studies phenotypically exploring the characteristics of human iPSC derived microglial cells harboring a CRISPR edited *FUS* ^*P525L*^ mutation identified FUS mislocalisation and transcriptomic alterations (Kerk et al., 2022). These included dysregulation in chemoreceptor signaling, cell motility, leukocyte chemotaxis, inflammatory response and cytokine production.

Chemoreceptor dysfunction was functionally validated through detection of exacerbated intracellular calcium signaling; however, no differences were found in the secretion of cytokines or phagocytosis, indicating a disconnect between transcriptional and functional phenotypes.

Transcriptomics has additionally been applied to human *C9orf72* mutant microglia, which display phenotypes including reduced C9orf72 protein, RNA foci and expression of dipeptide repeat proteins, however, separate studies have reported minimal changes in gene expression (Lorenzini et al., 2023; Vahsen et al., 2023). More gene expression changes in *C9orf72* mutant microglia were observed following treatment with lipopolysaccharides (LPS), an outer membrane component of gram‐negative bacteria commonly used to induce a pro‐inflammatory reactive state in microglia. LPS treatment of *C9orf72* mutant microglia resulted in an MMP9‐dependent increase in cytokine production and increased toxicity to co‐cultured motor neurons (Vahsen et al., 2023). Immunoprecipitation followed by mass spectroscopy in hiPSC derived microglia revealed that C9orf72 interacts with several regulators of autophagy (Banerjee et al., 2023). This finding led to experiments showing that *C9orf72* knockout in microglia disrupts autophagy initiation, leading to sustained inflammatory responses following LPS treatment and enhanced neurotoxicity of co‐cultured motor neurons undergoing excitotoxicity induced cell death.

hiPSC derived *C9orf72* mutant microglia with isogenic controls have been further utilized to explore findings originating from gene‐based rare variant analysis of whole genome sequencing data from the Project MinE ALS sequencing consortium (Eitan et al., 2022). The *IL18RAP* 3′UTR was identified as a rare variant significantly associated with protection against ALS, with certain variants reducing the disease odds ratio by around fivefold. The functional effects of this were explored by editing *C9orf72* mutant iPSC microglia with point mutations to mirror the most common protective *IL18RAP* variants, resulting in downregulation of the IL18RAP protein. Mass spectroscopy revealed that *IL18RAP* 3′UTR variants reduced the association of dsRNA‐binding proteins in microglia. The functional relevance of this was explored through co‐culturing control and mutant iPSC derived microglia stimulated with LPS and IL‐18 together with control hiPSC derived motor neurons, revealing that the *IL18RAP* 3′UTR variant containing microglia improved neuronal survival. RNA‐sequencing of microglia cells containing *IL18RAP* 3′UTR and treated with LPS and IL‐18 revealed downregulation of genes associated with pro‐inflammatory pathways including NF‐kB, TNF, NOD‐like receptor and Toll‐like receptor signaling. NF‐kB signaling inhibition resulted in a reduction of canonical *IL18RAP* microglial neurotoxicity, suggesting rare, protective variants increase microglia‐dependent motor neuron survival through a reduction in NF‐kB signaling.

Microarrays (Andrés‐Benito et al., 2017; Butovsky et al., 2015; Dangond et al., 2004; Ishigaki et al., 2002; Malaspina et al., 2001) and more recently bulk RNA‐seq analysis of post mortem ALS tissue (Brohawn et al., 2016; D'Erchia et al., 2017; Dols‐Icardo et al., 2020; Humphrey et al., 2023; Tam et al., 2019; Wang et al., 2021) have generally identified a pro‐inflammatory state as among the top dysregulated pathways in ALS post mortem tissue and implicated microglia‐expressed genes in ALS, among other glial signals. Bulk proteomics of synaptically enriched fractions from ALS cortical brains suggested increased expression of proteins involved in inflammatory pathways, potentially implicating aberrant microglial activity as being responsible for ALS synaptic alterations (Laszlo et al., 2022). Similarly, proteins increased in *C9orf72* mutant frontal cortex were in part involved in inflammatory processes (Umoh et al., 2018). In addition, the expression of microglial genes in human post mortem samples has been found to be correlated with more aggressive disease, although it is still not understood whether this reflects microglia driving disease, increased motor neuron degeneration resulting in an increased harmful reactivity of microglia or an attempted compensatory process (Humphrey et al., 2023). Gene co‐expression analysis has inferred cell type‐specific gene expression changes in post mortem ALS glia including increases in microglial enriched genes (Dols‐Icardo et al., 2020; Humphrey et al., 2023; Wang et al., 2021). However, gene expression changes may otherwise be attributable to cell‐type composition alterations of the spinal cord, as indicated in certain immunohistochemical studies (Spiller et al., 2018; Tam et al., 2019).

### 2.3 Oligodendrocytes

Oligodendrocytes, responsible for the formation of the myelin sheath surrounding neuronal axons in the CNS, among other homeostatic roles, are increasingly being studied in the context of ALS.

#### 2.3.1 Studies predominantly using mouse transgenic models of ALS

An increasing number of studies utilizing omics techniques are being applied to study oligodendrocyte ALS dysfunction, broadly identifying loss of homeostatic myelination function. For example, translational profiling of SOD1^G37R^ mutant oligodendrocytes, isolated using a Cnp1‐bacTRAP system, found at an early symptomatic time point after translational changes in motor neurons had begun, significant downregulation of genes encoding for proteins which comprise the myelin sheath (Sun et al., 2015). Concerning other ALS‐linked genes, oligodendrocyte‐specific deletion of *Tardbp* was shown to be important for oligodendrocyte survival and myelination and to have profound, cell maturation‐dependent effects (Heo et al., 2022; Wang et al., 2018). While differential gene expression in premyelinating oligodendrocytes was found to relate to mRNA processing and cell localisation (Heo et al., 2022), bulk RNA‐seq of spinal cords with *Tardbp* knockout specifically in oligodendrocytes revealed an upregulation of genes involved in the immune response and downregulation of genes involved in lipid metabolism, accompanied by demyelination and oligodendrocyte death (Ho et al., 2021). Loss of myelin was attributed to downregulation of Srebf2, a master regulator of cholesterol metabolism involved in myelin production (Horton et al., 2002).

#### 2.3.2 Studies predominantly using hiPSC models of ALS or human postmortem tissue

Postmortem studies have generally indicated a degeneration of oligodendrocytes through ALS disease progression (Kang et al., 2013). However, bulk RNA‐seq studies have found both increases (Wang et al., 2021) and decreases (Humphrey et al., 2023) of expression in oligodendrocyte associated genes in patient post‐mortem samples.

Recently, human iPSC derived oligodendrocytes harboring *FUS* R521H and P525L mutations have been generated (Zhu et al., 2024). RNAseq analysis displayed upregulation of genes relating to the cellular compartment ‘myelin sheath’. Comparison to a snRNAseq dataset from *C9orf72* patient motor cortex samples (Li, Jaiswal, et al., 2023) and RNAseq from a mutant *FUS* mouse model (Rossaert et al., 2019) proved contradictory to these differential gene expression changes as many oligodendrocyte genes relating to the myelin sheath were downregulated. Greater overlap was found with a RNAseq dataset from sporadic ALS patients (Tam et al., 2019). Lipidomics analysis revealed that myelin sheath perturbations may relate to lipid dysregulation identified in *FUS* mutant OPCs. By integrating both the lipidomics and transcriptomics datasets, dysregulated glycerophospholipid metabolism was identified in both *FUS* mutant lines. Analysis of FUS cross‐linking and immunoprecipitation data (CLIP) found that numerous RNA targets of FUS are involved throughout glycerophospholipid metabolism, many of which were identified in both *FUS* R521H and P525L mutant OPCs but also transgenic mice overexpressing wild‐type *FUS* and both sporadic and *C9orf72* patient samples.

As is evident from the above findings, clear concordance between individual studies is not always the case. The widespread adoption and utilization of bulk RNA sequencing across different ALS models may have built upon this dissonance, through discrepancies both between these omics datasets and many subsequent functional studies. Nonetheless, the continued effort to utilize and integrate ‘omics’ techniques, with particular focus on the functional recapitulation of findings, holds promise in building a comprehensive mechanistic understanding of glia within ALS.

## 3 CAPTURING INTERCELLULAR HETEROGENEITY USING SINGLE CELL OMICS METHODS

Our understanding of the potential cellular substate heterogeneity, which may be partly responsible for the aforementioned variability between studies and indeed patients, is increasingly being uncovered through single‐cell omics methods. Nonetheless, a key aspect of future studies will require the integration of findings across models and methods to provide insight into pathomechanisms and the identification of suitable therapeutic targets. The functional relevance of disease‐associated cellular substrates remains poorly understood and should be a key focus in future work (Clayton et al., 2024). Studies applying novel omics technologies to ALS patient samples have primarily focused on the differential response of neuronal subtypes to disease (Gautier et al., 2023; Pineda et al., 2024; Yadav et al., 2023). Shared consensus regarding sample processing and analysis pipelines and the integration between datasets, with further focus on glia, holds promise in decoding individual cell type contributions to disease progression.

### 3.1 Astrocytes

More recent single‐cell sequencing studies have further detailed the varied nature of astrocyte subpopulations in both health and disease. ScRNA‐seq of mutant *SOD1* ^*G93A*^ mouse pontine brainstem samples has further enabled better understanding of astrocyte subpopulations in ALS mouse models (Liu et al., 2020). Astrocytes displayed the largest amount of differentially expressed genes compared with other CNS cell types, with downregulated genes enriched in development and neurogenesis terms. SnRNA‐seq has also been performed on the lumbar spinal cords of *SOD1* ^*G93A*^ mutant mice (MacLean et al., 2022). Similarly astrocytes featured the greatest number of differentially expressed genes, which were primarily associated with complement activation, possibly as a result of an increased activity of transcription factors associated with inflammation and oxidative stress. In silico, *intracellular* networking inferred dysfunctional communication between motor neurons and glia at synapses.

Integrated sn‐RNAseq and snATAC‐seq of *C9orf72* mutant ALS frontal and motor cortex has recently identified transcriptional increases in astrocytes including reactivity and structural remodeling processes correlating with chromatin accessibility (Li, Jaiswal, et al., 2023). A subsequent study utilizing snRNA‐seq of not only *C9orf72* mutant but also sALS patient cortex samples, found broad and remarkably overlapping transcriptomic astrocyte reactivity and metabolic changes, highlighting the shared impact of astrocyte activation across a wide range of ALS patients (Pineda et al., 2024). Interestingly, previous findings using bulk RNA‐seq highlighting numerous microglial transcriptomics differences between C9orf72 and sporadic samples were not recapitulated (Humphrey et al., 2023), potentially due to technical constraints of either technique and high variability between patients and brain regions.

The effects of ALS astrocyte dyshomeostasis on neurons are increasingly being studied using organoids. Human organoid models have been generated to model cortical ALS pathology from human‐derived iPSCs harboring *C9orf72* mutations (Szebényi et al., 2021). scRNAseq revealed that glia and neuron composition and development did not differ substantially between control and mutant organoids. Comparison of differential gene expression changes did, however, reveal a number of differences within individual cell clusters, most notably within astrocytes and deep layer cortical neurons. Further analysis of differentially expressed genes utilizing GO analysis and weighted gene co‐expression analysis implicated enrichment in genes involved in astrocytic extracellular matrix remodeling, astroglia protein targeting, synaptic plasticity, and neuronal development. Transcription factor activity inference analysis predicted increases in the activity of transcription factors involved in ER stress, oxidative stress, unfolded protein response (UPR) activation and DNA damage. The increase in ER stress in *C9orf72* mutant organoids was further observed through increases in numerous proteins known to be positively associated with ER stress and UPR.

### 3.2 Microglia

ALS single‐cell studies capturing microglia have found a broad range of transcriptomic changes, however, the exact mechanism of microglial dysfunction remains unresolved. Outstanding questions include whether microglial phenotypes are different in specific mutant gene carriers compared with sporadic cases, and therefore occur partially cell autonomously, and if these precede and/or result from neurodegeneration, environmental cues or glial and immune cell interactions.

In a landmark study, spatial transcriptomics has been performed on *SOD1* ^*G93A*^ mutant mouse spinal cords at stages throughout disease to link gene expression changes to their location (Maniatis et al., 2019). Microglial dysfunction was found to be proximal to motor neurons and preceded symptom onset and astrocyte reactivity, reflecting previously suggested reactive astrocyte activation by microglia (Liddelow et al., 2017). scRNA‐seq of *SOD1* mutant mouse spinal cord has also been performed to identify disease associated microglia subpopulations (Mifflin et al., 2021). Three distinct populations were identified, with one cluster being annotated as ‘disease associated’ due to the high expression of *Apoe*, *Cd9*, *Trem2*, *Cd63*, *Cd52*, and *Ctsb*. Interestingly, this cluster showed significant overlap with previously published data from an AD mouse model (Keren‐Shaul et al., 2017). A second cluster was grouped as homeostatic microglia and a final cluster showed differentially expressed marker genes associated with inflammatory and NF‐κB regulated pathways. Treatment with a RIPK1 inhibitor reduced the size of this final inflammatory cluster, increasing the number of designated ‘homeostatic’ microglia. While scRNA‐seq identified changes in subpopulations of spinal cord microglia, scRNA‐seq of *SOD1* ^*G93A*^ mouse cortex revealed only subtle changes in microglial transcriptomes limited to later disease stages (Filipi et al., 2023).

The application of both bulk and scRNA‐seq of microglia isolated from *C9orf72* knockout mice has found increases in interferon genes (*Isg15*, *Ifit27*, *Oas1a*, *Stat2*) and decreases in homeostatic (*P2ry12*, *Ccr5*, and *Gpr34*) and activated response (*Clec7*, *Cst7*, *Spp1*) genes (Lall et al., 2021). While scRNA‐seq confirmed the changes observed using bulk RNA‐seq, no differences were found in the proportions of different microglial populations in *C9orf72* knockout microglia, possibly due to a lack of motor neuron degeneration in this model. A subsequent study found broad overlap and largest differentially expressed gene signatures in endothelial, microglial and astrocyte populations in C9orf72‐deficient mice (Limone, Couto, et al., 2024). Many of these, known to be dysregulated in ALS, related to antigen presentation and oxidative phosphorylation, among other pathways, and were similarly dysregulated in peripheral immune cells. When C9orf72‐deficient mice were treated with an IL‐17a neutralizing antibody, numerous markers of inflammation were found to be reduced, in addition to improvements in motor function. However, it is noteworthy that this model does not exhibit classical motor neuron degeneration. Therefore, it follows that certain microglial phenotypes that are a consequence of neurodegeneration will not be observed.

More recently, snRNA‐seq has been applied to sALS patient cortices (Limone, Mordes, et al., 2024). Alongside upregulation of cellular stress genes in excitatory neurons, microglial upregulation of genes relating to endocytosis, exocytosis and lipid metabolism previously associated with familial ALS‐FTD was found. *In vitro* co‐culture of apoptotic neurons and human embryonic stem cell derived microglia mirrored some of these transcriptomic changes, suggesting the observed indicative microglial reactive state may be in response to neuronal apoptosis and not necessarily cell autonomous. Further to this, snRNA‐seq of sporadic and *C9orf72* mutant ALS‐FTD patient motor and prefrontal cortices similarly found specific populations of excitatory neurons vulnerable to disease, in addition to a general transcriptomic shift reflecting loss of microglial homeostatic function (Pineda et al., 2024). Contrary to findings in C9orf72 deficient mouse models (Lall et al., 2021), microglia featured a downregulation of known inflammatory response pathway terms and other reactive microglia markers such as *APOE*, *CD14* and *CD86*, possibly due to both interspecies differences but also the differential effects of the *C9orf72* repeat expansion found in human patients compared with *C9orf72* deficient mice.

## 4 THE FUTURE OUTLOOK OF OMICS IN ALS GLIAL BIOLOGY AND BEYOND

An overarching goal in ALS is the development of a cure, a prerequisite to which is a precise understanding of pathogenesis. However, due to the intricate nature of the disease's pathophysiological mechanisms, which involve complex interactions among internal and external factors, it is likely that we have only scratched the surface of what remains to be discovered. Historically, glial cells were seen as having a secondary role in ALS pathology; however, the use of omics approaches has revealed that this is not the case. It is now evident that glial cells not only are prominent actors but could be the missing pieces of the puzzle. We anticipate that the coming years will witness a surge in studies focused on ALS glial biology, leading to significant advancements in our understanding of the disease and, ultimately, more impactful disease‐modifying therapies. Specifically, we foresee that the adoption of new omics methods and, crucially, the rational integration of multiple omics methods will catalyze translational science in this context, as alluded to below.

Understanding a system in its entirety is not achievable by examining its individual components in isolation, and the same principle applies to deciphering the role of glial cells in ALS. Relying solely on a single omics approach provides a limited perspective, often excluding crucial other aspects of the disease. While we have a wide array of technologies and models at our disposal to comprehend the role of glia in ALS, it is evident that the primary focus has largely revolved around using bulk RNA sequencing and proteomics. This emphasis can be attributed to the pace of technological advances and barriers to their widespread adoption (e.g., resource, expertise etc.). With time, there has been a reduction in cost and complexity of these newer methods, which will inevitably lead to a surge in their adoption. There are clear examples discussed above including scRNA sequencing and spatial transcriptomics (Liu et al., 2020; MacLean et al., 2021; Maniatis et al., 2019). Another promising avenue lies in the exploration of non‐coding RNAs, which were once overlooked in disease pathogenesis but are now recognized for their crucial roles (Men et al., 2019; Morel et al., 2013; Simone et al., 2021). The incorporation of technologies like microRNA sequencing and total RNA sequencing will complement single‐cell techniques and yield new insights. Additionally, epigenomic and epitranscriptomic modifications, encompassing DNA and RNA methylation as well as histone modifications, play a pivotal role in gene regulation. Although these methods have been utilized in ALS motor neurons (Li, Dou, et al., 2023; McMillan et al., 2023), they remain relatively unexplored in glial cells. Metabolomics has the capacity to discern metabolic signatures linked to disease progression or response to treatment. For example, lipidomic analysis of *FUS* mutant mice has found no changes at the presymptomatic stage, unlike *SOD1* ^*G93A*^ mutant mice (Cutler et al., 2002; Dodge et al., 2015), however revealed widespread lipid dysregulation at the symptomatic stage (Burg et al., 2021). Integration of lipidomic and RNA‐seq datasets found gene dysregulation of key enzymes associated with glycerophospholipid metabolism potentially due to changes in their respective transcription factors through altered FUS function.

The use of omics to discover biomarkers holds the potential to significantly advance early diagnosis and monitoring of ALS. Other, more intricate and bespoke, technologies are also noteworthy in this context. For example, identifying the RNA targets of a given RNA binding protein within glia (methods such as eCLIP, iCLIP, and PAR‐CLIP [Danan et al., 2016; Konig et al., 2011; Van Nostrand et al., 2016]). The concept of RBPome capture focuses on the study of isolating RNA‐binding proteins bound to target RNA molecules, thus shedding light on the post‐transcriptional regulation mechanisms within cells. Given the importance of RBPs in the physiopathology of ALS, it is imperative not to disregard this sub‐category of omics.

The adoption of multi‐omics strategies is gaining traction, both generally and also in the context of ALS. As an example, a recent study applied a combination of omics strategies together with machine learning; from a cohort of heterogeneous samples with different familial ALS mutations, they identified shared pathogenic signatures leading to the convergent cellular phenotypes (Catanese et al., 2023). Similarly, a subsequent study found that during motor neuron development, an increase in m6A RNA methylation redirects the function of an RNA‐binding protein towards a specific subset of transcripts, consequently regulating their protein expression level. This study encompassed transcriptomics, proteomics, epitranscriptomics, and targetome analysis for a particular RBP. This comprehensive strategy revealed the temporal convergence of multi‐layered gene regulation, which would have been challenging without the application of multi‐omics technologies (Klein et al., 2024). One can envisage fully employing the omics technologies at our disposal in a single study on glia, but with such staggeringly rich datasets comes an equally large challenge of data analysis and integration. Integrating a variety of different types of datasets, interpreting their biological significance amidst the abundance of information, and managing the computational resources can be perceived as significant obstacles and clearly necessitate strategic investment in data science. Effective approaches to reveal commonalities between datasets include meta analysis and integrated analysis. Indeed, the latter has been applied to transcriptomic data from 429 iPSC‐derived human motor neuron datasets, and 271 post‐mortem spinal cord samples to reveal genome instability and its link to TDP‐43 pathology (Ziff et al., 2023). As more omics datasets emerge from ALS glia, similar analytical pipelines can be applied to refine our perspective of salient pathways, which can be orthogonally validated and mechanistically evaluated. These approaches will enable us to scrutinize the diversity within glia in ALS at an unprecedented level, leading to the identification of distinct cell subtypes, expression patterns, and functional roles.

## 5 CONCLUDING REMARKS

The rapidly increasing generation of advanced omics undoubtedly raises the question of how to effectively integrate and analyze these datasets to uncover the true essence of the disease. This challenge extends beyond the context of glia and encompasses the broader scope of ALS. Over the past few years, there has been a surge in studies harnessing machine learning, which possesses the capability to provide comprehensive understanding of a disease at the molecular level and uncover hidden patterns (Hagemann et al., 2021; Verzat et al., 2022), predict outcomes, drive the development of diagnostics tools, identify new biomarkers and guide therapeutic design. Nonetheless, it is crucial to recognize that machine learning presents substantial obstacles. While exploring these challenges is outside the scope of this study, it is worth acknowledging that they play an integral role in preventing the integrating of multi‐omics techniques. In this age of big data, we can foresee a time not too distant when the primary focus shifts away from generating datasets in ALS glia. Instead, the upcoming phase will emphasize democratizing the utilization of machine learning.

This progression may ultimately pave the way for an absolute understanding of the disease, indispensable in our quest to find a cure.

Important considerations in future ALS glial studies remain establishing primacy (inciting molecular events), salience (importance of these events in the overall disease cascade) and identifying which events are viable therapeutic candidates through established approaches (e.g., small molecular pharmacology or antisense oligonucleotides). Human stem cell differentiation into clinically salient glial subtypes coupled with multi‐omic approaches represent an exciting prospect for addressing these above‐mentioned issues. Shared rationale regarding experimental design, including the use of isogenic controls and the necessity of utilizing multiple cell lines from a representative and wide genetic background, will be imperative in ensuring reproducible results upon which shared consensus may be built. Further to this, studies should be orthogonally validated in animal models and/or human post‐mortem tissue. Following data integration and analysis, candidate pathways can be tested using high‐throughput screening methods for their capacity to ameliorate disease‐specific phenotypes. This approach also offers promise in terms of toxicity assays, for example ensuring that a small molecule found to ameliorate astrocyte harmful reactive transformation does not cause adverse effects for neurons or microglia. Cumulatively, these approaches offer real hope for precision medicine.

## AUTHOR CONTRIBUTIONS

S.M wrote the first draft of the manuscript with editing from PK, SB, BC and RP. All authors have read and approved the final manuscript.

## Data Availability

Data sharing is not applicable to this article as no new data were created or analyzed in this study.
